# Nonconsensual withdrawal of nutrition and hydration in prolonged disorders of consciousness: authoritarianism and trustworthiness in medicine

**DOI:** 10.1186/1747-5341-9-16

**Published:** 2014-11-07

**Authors:** Mohamed Y Rady, Joseph L Verheijde

**Affiliations:** 1 Department of Critical Care Medicine, Mayo Clinic Hospital, Mayo Clinic, Phoenix, Arizona, USA; 2 Department of Physical Medicine and Rehabilitation, Mayo Clinic, Scottsdale, Arizona, USA

**Keywords:** Dehydration, Euthanasia, General anesthesia, Minimally conscious state, Opioids, Prolonged disorders of consciousness, Sedatives, Unresponsive wakefulness syndrome, Vegetative state

## Abstract

The Royal College of Physicians of London published the 2013 national clinical guidelines on prolonged disorders of consciousness (PDOC) in vegetative and minimally conscious states. The guidelines acknowledge the rapidly advancing neuroscientific research and evolving therapeutic modalities in PDOC. However, the guidelines state that end-of-life decisions should be made for patients who do not improve with neurorehabilitation within a finite period, and they recommend withdrawal of clinically assisted nutrition and hydration (CANH). This withdrawal is deemed necessary because patients in PDOC can survive for years with continuation of CANH, even when a ceiling on medical care has been imposed, i.e., withholding new treatment such as cardiopulmonary resuscitation for acute life-threatening illness. The end-of-life care pathway is centered on a staged escalation of medications, including sedatives, opioids, barbiturates, and general anesthesia, concurrent with withdrawal of CANH. Agitation and distress may last from several days to weeks because of the slow dying process from starvation and dehydration. The potential problems of this end-of-life care pathway are similar to those of the Liverpool Care Pathway. After an independent review in 2013, the Department of Health discontinued the Liverpool Care pathway in England. The guidelines assert that clinicians, supported by court decisions, have become the final authority in nonconsensual withdrawal of CANH on the basis of “best interests” rationale. We posit that these guidelines lack high-quality evidence supporting: 1) treatment futility of CANH, 2) reliability of distress assessment from starvation and dehydration, 3) efficacy of pharmacologic control of this distress, and 4) proximate causation of death. Finally, we express concerns about the utilitarian-based assessment of what constitutes a person’s best interests. We are disturbed by the level and the role of medical authoritarianism institutionalized by these national guidelines when deciding on the worthiness of life in PDOC. We conclude that these guidelines are not only harmful to patients and families, but they represent the means of nonconsensual euthanasia. The latter would constitute a gross violation of the public’s trust in the integrity of the medical profession.

## 

“A society that believes in nothing can offer no argument even against death. A culture that has lost its faith in life cannot comprehend why it should be endured.”

Andrew Coyne 1994

## Introduction

The Royal College of Physicians (RCP) of London published national clinical guidelines on prolonged disorders of consciousness (PDOC) in 2013 to standardize the approach to diagnosis, management, and end-of-life care (EOLC) for 2 specific neurologic disorders: vegetative and minimally conscious states [1]. Jennett and Plum [2] introduced the term *vegetative* based on the assumption that the remaining neurologic processing is limited to only the brainstem. Forty years later, higher cortical neurologic processing is widely acknowledged to be retained in what thus far has been termed the *vegetative state*. Therefore, this phrase has been replaced in the neuroscientific literature with a more scientifically precise descriptor, *unresponsive wakefulness syndrome*[3]. The persistent use of *vegetative* in the medical literature may reflect a fundamental misunderstanding of this neurologic disorder or perhaps connote an indignity to persons with development of this neurologic disability.

Refusal of nutrition and fluid by competent adults is a legal method of suicide and assisted death [4]. Administration of nutrition and hydration, even by artificial means, is considered by many authors to be standard care rather than medical treatment [5-15]. Denton et al. have made the argument along a physiological line of reasoning that nutrition and hydration alleviate natural responses of hunger and thirst which are primordial emotions of evolutionary origin in human consciousness [16]. However, the RCP national guidelines [1] use the term *clinically assisted nutrition and hydration* (CANH) to emphasize that the assistance should be considered a medical treatment rather than a basic compassionate care service rendered to disabled persons. Patients in PDOC lack decision-making capacity and cannot provide first-person consent for a life-ending intervention such as stopping nutrition and hydration. These patients can survive for years with CANH, even if a “ceiling of care” is implemented, i.e., withholding new interventions for acute life-threatening illness such as infections, thromboembolism or acute cardiopulmonary arrest (p. 70) [1]. If patients in PDOC do not improve with neurorehabilitation, the treating clinicians can authorize cessation of nutrition and hydration. CANH is withdrawn based on the determination of treatment futility and unacceptable quality of life. The guidelines apply the “best interests” rationale when justifying nonconsensual withdrawal of CANH.

This commentary focuses on the medical and ethical issues preempting nonconsensual withdrawal of CANH and the use of a preferred end-of-life care (EOLC) pathway (Table 1) [1]. The EOLC pathway is centered on a staged escalation of medications, including sedatives, opioids, barbiturates, and general anesthesia, and it has the same problems as those reported with the Liverpool Care Pathway [17]. The Department of Health has discontinued the Liverpool Care Pathway in England after an independent review in 2013 [18]. We question the validity of treatment futility of CANH on the basis of several factors: 1) the contemporary knowledge gap about the timeline for recovery in PDOC, 2) the pathophysiology of distress from dehydration, 3) the efficacy of administered medications in managing this distress, and 4) the proximate cause of death from lethal effects of these medications. We also address the predominantly utilitarian interpretation of best interests in the justification of nonconsensual and terminal withdrawal of CANH in PDOC.

**Table 1:** **National guidelines for the Staged Escalation of pharmacologic management of distress from the withdrawal of nutrition and hydration in prolonged disorders of consciousness**^**a**^

Stage 1: Continuous IV infusion of benzodiazepines and opioids	Medications are best loaded in separate syringe drivers so that they can be varied independently until the optimum regimen is established. Set up 2 IV syringe drivers and commence IV infusion with midazolam (10 mg/24 h) and morphine (10 mg/24 h). Prescribe bolus IV doses of each drug to be given by the syringe pump. Adjust the infusion dose according to the frequency of bolus doses required (midazolam, up to 10–20 mg/h; morphine, 10 mg/h). However, if no effect is seen from bolus doses, the patient is receiving the maximum benefit from these drugs. Progress to stage 2.
Stage 2: Continuous IV infusion of neuroleptics	Continue the current doses of morphine and midazolam in 1 IV syringe driver. Set up a second syringe driver with levomepromazine (50 mg/24 h). Prescribe bolus IV doses of levomepromazine (12.5-25 mg). However, if no effect is seen from bolus doses, progress to stage 3.
Stage 3: Continuous IV infusion of barbiturates	Continue morphine and midazolam at current dose in first continuous IV infusion. Stop levomepromazine. Replace with phenobarbitone (600 mg/d) in a second continuous IV infusion. Prescribe phenobarbitone (100–200 mg) IV bolus doses. If not responding to bolus doses, proceed to stage 4.
Stage 4: General (self-ventilating) anesthesia	In very rare cases, severe physiologic distress with terminal agitation may require self-ventilating IV anaesthesia. This should be administered with the support of ITU-trained staff under the supervision of a consultant anaesthetist.

*Abbreviations*: *ITU* intensive therapy unit, *IV* intravenous.

Reproduced from: Royal College of Physicians. Prolonged disorders of consciousness: National clinical guidelines. London: RCP, 2013 (p. 84) [1]. Copyright © 2013 Royal College of Physicians. Reproduced with permission.

^a^The table illustrates the staged escalation of benzodiazepines, opioids, neuroleptics, and general anesthesia in the end-of-life care pathway after the withdrawal of nutrition and hydration in prolonged disorders of consciousness.

## Knowledge gap in the understanding of disorders of consciousness

**Figure 1:**
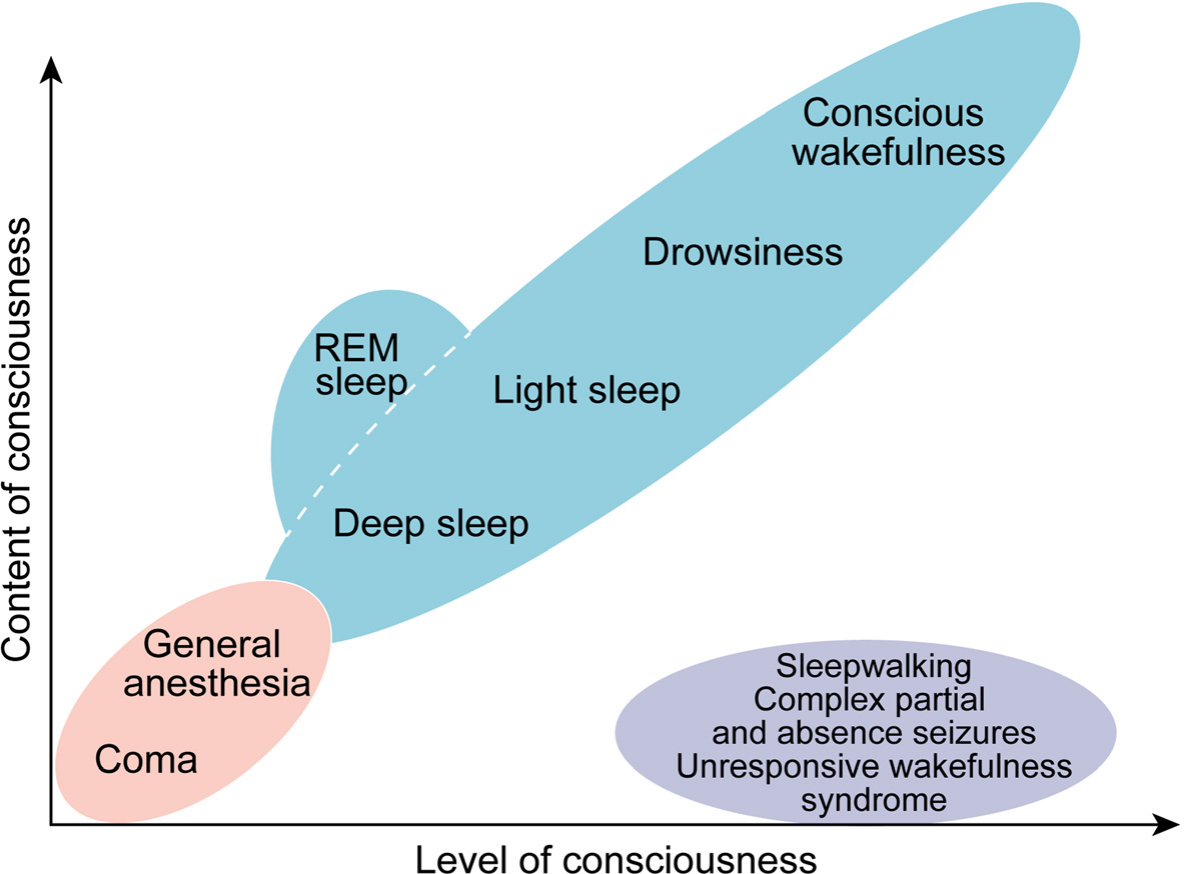
**The interrelationship between the level and the content of consciousness in different pathophysiologic and pharmacologic states.** Contemporary advances in neuroscience have unmasked a wide knowledge gap in the neurophysiologic characterization of human consciousness. The level of consciousness is generally assessed by either wakefulness or responsiveness to external stimuli. The content of consciousness includes internal (self) and external (environmental) awareness. The content of consciousness is difficult to assess in unresponsive and noncommunicative patients (e.g., coma or general anesthesia settings). The temporal pattern of recovery in neuronal networks that mediate the content of consciousness in the severely injured human brain has not been completely elucidated. Adapted from Laureys [20] with permission of the publisher Elsevier Ltd.

Advances in neuroimaging and neuroelectrophysiologic monitoring have unmasked a large knowledge gap in the contemporary understanding of consciousness [19]. Neuroscientific knowledge of the interrelationship between the level and the content of consciousness (i.e., responsiveness and awareness, respectively) in different pathophysiologic and pharmacologic states continues to evolve (Figure 1) [20-23]. Functional neuroimaging and neurophysiologic studies of the injured human brain suggest remarkable plasticity of neural connectivity and networks involved in external and internal awareness [24,25]. This also highlights the potential for recovery and retention of awareness, in spite of extensive brain injury [19]. However, the optimal neurotherapeutic modalities and timelines for recovery of awareness and responsiveness in PDOC have not been fully characterized and continue to evolve with new advances in neuroscience [26].

The RCP guidelines set an arbitrary time limit of 4 weeks to 6 months for making end-of-life decisions in PDOC (p. 77) [1]. End-of-life decisions are made on the basis of a clinical assessment showing lack of improvement despite neurorehabilitation. This assessment is generally limited to a bedside neurologic examination and may not include sensitive tools for assessing awareness such as functional neuroimaging and neurophysiologic studies [24,25]. When end-of-life decisions are made prematurely because of pervasive “therapeutic nihilism”, this becomes a self-fulfilling prophecy for poor patient outcomes [27].

## Pathophysiology of life-ending starvation and dehydration

The RCP guidelines delineate some of the symptoms induced by prolonged starvation and dehydration: “[t]he consequence of treatment withdrawal would be death by dehydration and nutritional deprivation with the patient possibly experiencing (i) thirst, (ii) hunger, (iii) discomfort, (v) [*sic*] distress, and (vi) pain for a 2–3 week period” (p. 78) [1]. The distress includes agitation, sweating, and “spontaneous and reflex movements such as roving eye movements, groaning, crying, teeth-grinding, chewing etc. which may become more pronounced” (p. 80) [1].

The guidelines claim that the intention of withdrawing CANH is to avoid inflicting additional suffering. However, we posit that the guidelines downplay the distress and suffering from intentional starvation and dehydration by asserting that the benefits of withdrawal outweigh the associated short-term harm. In clinical practice, it is generally accepted that CANH is unlikely to cause pain or suffering unless patients are intolerant of enteral feeding because of abdominal distention, vomiting, or fluid overload. Most patients in PDOC are unlikely to be intolerant of nutrition and hydration. In fact, the literature suggests (and most families agree) that nutrition and hydration are considered basic compassionate care because they promote physical and emotional well-being [10,28,29].

Withdrawal of CANH has biologic consequences [30]. The lethal pathophysiology of intentional dehydration includes onset of acute kidney failure and cardiovascular collapse. However, this dying process is slow and can last days to weeks. The physiologic responses to starvation and dehydration in the dying process also diminish the efficacy of analgesics and sedatives in controlling distressful symptoms [31,32]. Furthermore, the adverse effects of these medications (e.g., myoclonus, agitation, delirium, hallucination, hyperalgesia, seizure, paralytic ileus, cardiorespiratory depression, etc.) are potentiated by dehydration [30]. Optimal pharmacologic control of these symptoms becomes clinically challenging in the last few days of life. Indeed, the guidelines resort to using barbiturates and general anesthesia to control refractory distress in the final days before death (Table 1).

The neurologic consequences of withdrawal of CANH also are not completely appreciated. Without appropriate neuromonitoring, bedside clinical assessment cannot easily determine awareness of internal and external noxious stimuli. Patients who are rendered pharmacologically unresponsive with general anesthesia retain primary sensory processing of noxious stimuli [33]. Awareness of primordial affective responses to thirst, hunger, and pain are normally mediated through higher and lower brain structures [16]. These affective responses are intensified in PDOC [34]. Although the guidelines recognize that higher neurologic processing of noxious stimuli from prolonged starvation and dehydration can produce physical signs “e.g., grimacing, moaning, etc.” (pg. 60) [1], they do not emphasize that absent signs do not exclude central nociception or affective distress.

## Efficacy of sedation in managing distress of dehydration

Sedatives and opioids are administered preemptively to manage distress after the withdrawal of life-sustaining treatment [35]. These medications can be used to induce and maintain continuous deep sedation until death [31]. Continuous deep sedation until death is also used as an alternative to active euthanasia [36-41]. The RCP guidelines prescribe a pharmacologic protocol for managing the distress induced by starvation and dehydration (Table 1). The protocol is centered on the delivery of escalating doses of midazolam and morphine by continuous subcutaneous or intravenous infusion, with no proportionality in dose titration: “[n]ever decrease the background infusion dose, even when symptoms/signs appear to be well controlled” (p.82) [1]. However, dehydration alters the pharmacokinetics and pharmacodynamics of midazolam and morphine that are administered by continuous infusion. Accumulation of metabolites of both medications is lethal and causes cardiorespiratory arrest and death. A continuous infusion of barbiturates is a potent depressant of vital functions of the central respiratory center and the cardiovascular system. For the same reason, these medications can be administered rapidly as a bolus injection rather than by slow continuous infusion in active euthanasia and execution by lethal injection [42]. The RCP guidelines recommend general anesthesia to be administered in some cases “under the supervision of a consultant anaesthetist” (p. 84) [1]. The administration of general anesthesia without cardiopulmonary support is inconsistent with the standard of practice by anesthetists since “*their actions could be construed as an act of euthanasia or assisted suicide* as they would be initiating cardiopulmonary compromise and yet be unable to treat this once it had occurred” [emphasis added] [43].

The guidelines’ recommended pharmacologic protocol has not been validated in well-designed controlled clinical trials to ascertain its palliative efficacy in patients who are dying from prolonged starvation and dehydration. Authors of a Cochrane review expressed concerns regarding the safety of EOLC pathways that are formulated by opinions and without supporting high-quality evidence [44]. The implementation of this pathway in clinical practice can have harmful outcomes on dying patients, families, and health care providers and professionals [18,45-47]. A minimal standard is required in the formulation of safe and trustworthy national and international clinical practice guidelines [48]. The strength of evidence supporting a specific recommendation and the adaptability to an individual patient’s care goals, values, and preferences are required elements to ensure the guidelines are deserving of the public’s trust [49]. Notably, European palliative care experts consider sedation for intentional starvation and dehydration as euthanasia, not palliative care [50]. The World Health Organization describes palliative care as “relief from pain and other distressing symptoms” [51]. No high-quality evidence substantiates the efficacy of opioids and sedatives to control the distress associated with starvation and dehydration. Lacking such evidence, the withdrawal of CANH should not be considered palliative care in PDOC.

## Cause of death

The newly established guidelines state that “the principal process in the death is multi-organ failure from dehydration” (p.80) [1]. The proximate cause of cessation of vital signs is dehydration and the lethal cardiovascular and respiratory effects of the administered medications [31]. However, the guidelines recommend “[w]hen drawing up a death certificate after withdrawal of CANH, the original brain injury should be given as the primary cause of death” (p.84) [1]. Selecting the original brain injury as the primary or proximate cause of death provides legal sanctuary for clinicians who participate in the withdrawal of CANH. However, the discordance between the proximate cause of death and the official cause listed on the death certificate also could be interpreted as an infringement on the long-held assumption in society and law that the medical profession holds truthfulness as one of its highest moral priorities.

The UK courts have avoided adjudicating if nonconsensual withdrawal of treatment and administration of “potentially fatal doses” of palliative medications to hasten death is homicide [52]. Indeed, withdrawal of CANH fulfills two elements of homicide as outlined by South African Law Professor McQuoid-Mason [52]. He quotes from ‘Mason and McCall Smith’s Law and Medical Ethics’ that “[t]he courts in the UK ‘have been anxious to ensure that the cause of death was attributed to natural disease in all these cases of nonvoluntary assistance in dying [53]”. Firstly,”… ‘[e]ventual intention’ occurs where a person does not mean to kill a person but subjectively foresees the possibility of death because of their conduct and proceeds with such conduct” [52]. The eventual intention of withdrawing CANH is death. Secondly, “[l]egal causation occurs where the act or omission that caused the death is either a foreseeable or a direct cause of the person’s death. The foreseeability approach holds that if a person in the position of the perpetrator would have reasonably foreseen the likelihood of death and persisted with their act or omission, then the perpetrator legally caused the death of the deceased.…. The direct consequence approach holds that the perpetrator is liable unless some new act intervenes between the original act or omission that resulted in the ultimate death of the deceased… The victim’s pre-existing physical susceptibilities are not regarded as a new intervening act” [52]. Therefore, the preexisting physical disability in PDOC does not negate that the act of withdrawal of CANH is the proximate cause of death.

## The Utilitarian “Best Interests”

The RCP guidelines apply the best-interests standard in the justification of nonconsensual withdrawal of CANH after considering 3 pertinent issues: 1) the suffering induced by dehydration, 2) the cause of death, and 3) the value of human life. As we have outlined above, the guidelines fail to provide high-quality evidence that the pharmacologic interventions will consistently and effectively control distress from dehydration lasting for days to weeks. We have also argued that the proximate cause of death is dehydration and lethal effects of the administered medications. Finally, in what follows, we address how the guidelines transform best interests to justify nonconsensual life-ending withdrawal of CANH.

The guidelines do not consider the sanctity of human life as an absolute principle in law and perhaps in society at large: “[a]lthough the fundamental principle of law is the sanctity of human life, this is not an absolute principle. Life does not have to be prolonged regardless of circumstances” (p.62) [1]. The standard of best interests is applied in the determination of the value of a human life. The guidelines equate the value of life with quality of life, e.g., patients in PDOC without demonstrated neurologic improvement after a predefined period of neurorehabilitation have lives considered unworthy of prolonging: 1) “[b]est interests are not restricted purely to medical considerations, nor do they necessarily mean the prolongation of life” (p. 54); 2) “A formal best interests decision meeting should normally be held at least within 4 weeks after the onset of PDOC” (p. 58); and 3) “Once it is known that a patient is in a permanent VS [vegetative state], further treatment is considered futile. Processes to consider withdrawal of life-sustaining treatments, including CANH, should begin on the basis of their best interests, and in discussion with the family” (p. 76) [1].

The guidelines outline the balance of benefits and burdens of CANH (p. 78) [1]. The benefit of CANH is maintaining wellbeing through nutrition and hydration, continuation of life, and future improvement in the quality of life. The burdens of CANH include “potentially negative aspects of continuing life from physical or emotional discomfort from neurorehabilitation, pain or discomfort from frequent replacement of feeding tubes, vomiting, aspiration, lack of dignity, etc.” (p. 78) [1]. The guidelines then conclude that the burdens outweigh the benefits and determine that CANH is a futile treatment in PDOC and should be withdrawn based on best-interests considerations.

Surveys of families of patients in PDOC indicate that a significant number of them would object to withdrawing CANH [54,55]. Most families consider death by starvation and dehydration an “inhumane” [54] and “utterly abhorrent” [55] practice. Those families do not consider CANH futile because it is physiologically effective in maintaining nutrition and hydration for their loved ones. Anticipating the potential for familial refusal to life-ending withdrawal of CANH, the guidelines provide a roadmap of how to overcome this barrier: “[i]t should be made clear that a decision made in a person’s ‘best interests’ is not necessarily the same as the whole family being happy about a particular decision (for example, a family cannot easily be expected to say that they ‘want’ or ‘are happy’ to allow death)” (p. 57) [1].

Clinicians are authorized to withdraw CANH after an assessment and judgment of the patient’s best interests: “the responsible senior clinician has ultimate responsibility for healthcare decision-making based on judgement of what is in the patient’s best interests, taking into account what the patient would want if they could express a view” (p. 56) [1]. However, if treating clinicians object to the withdrawal of CANH, then the guidelines classify this objection as “conscientious objection” that “may not, of course, be well informed”, (p. 65) [1]. Objecting clinicians are to be replaced by clinicians who will carry out this decision: “[h]owever, if the individual clinician could not sanction best interests decision in one direction, they should hand over the care of the patient to a clinician who can” (p. 66) [1]. The default direction in best interests is the withdrawal of CANH. Finally, the Court system may be involved to sanction life-ending withdrawal of CANH: “a formal best interests decision-making meeting between the treating team, the family, and the responsible commissioning health body to decide whether it is appropriate to make an application to the Court for withdrawal of CANH, based on the balance of best interests, considering the benefits and burdens of continued treatment” (p. 79) [1].

The guidelines expect the legal system to concur with clinicians’ interpretation of best interests and proceed with nonconsensual withdrawal of CANH. The legal system can also trump objections to withdrawal by a Health and Welfare Lasting Power of Attorney or a Court-appointed Welfare Deputy. The guidelines point out that the court has ruled in Airedale NHS Trust v Bland [1993] AC 789 (HL) to authorize the withdrawal of CANH in vegetative states. The ruling asserts that nutrition and hydration are to be considered treatment rather than a basic compassionate care service. However, in the case of Bland (who was in a vegetative state), his family considered him to be already “dead” and requested withdrawal of CANH [56]. Therefore, withdrawal of CANH did not need to be invoked unilaterally by either clinicians or the court.

The guidelines appear to extrapolate this court ruling as an endorsement of nonconsensual withdrawal of CANH in all patients with a similar diagnosis. This example of medical authoritarianism should be troubling to both the majority of the medical community and to the general public. Special-interests groups have used the legal system to legitimize the controversial utilitarian practice of euthanasia, a practice that many believe violates traditional values of medicine and society [57]. Indeed, some members of the RCP working party, who developed the guidelines, have already called for active euthanasia of patients in permanent vegetative state [58]. Life-ending starvation and dehydration is becoming a common practice as an alternative to euthanasia by lethal injection in several European countries [12,14,59]. The Council of Europe has also endorsed the practice in EOLC guidelines [60]. This unconditional endorsement is inconsistent with the obligation of protecting rights of the vulnerable and incapacitated individuals in society. It raises the question —is society willing to sanction clinicians terminating “life that is unworthy of living” under the pretext of relieving societal burden and best interests?

## Conclusions

The national guidelines’ recommendation of life-ending withdrawal of nutrition and hydration after a finite time and implementation of staged escalation of potentially lethal medications in PDOC is not supported by high-quality evidence and is likely to harm patients and families. The utilitarian legal interpretation of best interests to sanction nonconsensual withdrawal of CANH risks paving the way to embracing nonconsensual euthanasia in medicine and society.

## Abbreviations

CANH: Clinically assisted nutrition and hydration; EOLC: End-of-life care; PDOC: Prolonged disorders of consciousness; RCP: Royal College of Physicians.

## Competing interests

The authors declare that they have no competing interests.

## Authors’ contributions

MYR and JLV have made substantial contributions in drafting the manuscript and revising it critically for important intellectual content, that they have given final approval of the version to be published, and that they have participated sufficiently in the work to take public responsibility for appropriate portions of the content. Both MYR and JLV have read and approved the final manuscript.

## Authors’ information

MYR: Consultant, Department of Critical Care Medicine, Mayo Clinic Hospital, Phoenix, Arizona; and Professor of Medicine, Mayo Clinic College of Medicine. JLV: Assistant Professor , Department of Physical Medicine and Rehabilitation, Mayo Clinic, Scottsdale, Arizona; and Associate Professor of Biomedical Ethics, Mayo Clinic College of Medicine.
